# Expanding equity horizons in knowledge sharing: How can global health journals level up?

**DOI:** 10.3389/ijph.2026.1609678

**Published:** 2026-05-01

**Authors:** Shashika Bandara, Carmen Sant Fruchtman, Nandi Joubert, Lucienne Zinsstag, Ananya Banerjee, Isabel Munoz Beaulieu, Moses Tetui, Vivian Qiang

**Affiliations:** 1 Department of Global and Public Health, School of Population and Global Health, McGill University, Montreal, QC, Canada; 2 Department of Epidemiology and Public Health, Swiss Tropical Public Health Institute, Allschwil, Switzerland; 3 University of Basel, Basel, Switzerland; 4 Department of Epidemiology, Biostatistics, Occupational Health, School of Population and Global Health McGill University, Montreal, QC, Canada; 5 Department of Family Medicine, McGill University, Montreal, QC, Canada; 6 School of Public Health Sciences, University of Waterloo, Waterloo, ON, Canada

**Keywords:** academic publishing, epistemic justice, global health, governance, knowledge sharing

## Introduction

In the past decade, the global health community has recognized and increasingly challenged unfair knowledge practices to address epistemic injustice within the field [1, 2]. Bhakuni and Abimbola frame epistemic injustice as “pervasive wrongs related to knowledge production, use, and circulation in global health.” [2] Structural barriers within academic publishing have resulted in perpetuating harmful power hierarchies in knowledge creation and sharing–dictating who is seen as an expert, and whose knowledge is prioritized and recognized as valid [2, 3]. These structural barriers include high article processing charges (APCs), paywalls that limit access, dominance of English as the language of publication, funding that is disproportionately concentrated in, or routed through Global North institutions [1]. Despite identifying these barriers, we have missed many opportunities to systemically change the global health academic publishing landscape [4].

Building a counterculture to dismantle structural barriers perpetuating epistemic injustice continues to be an uphill battle. Estimates in 2024 show that profit margins of academic publishing were as high as 40%, surpassing even silicon-valley tech companies such as Google [5]. While current efforts include journals restructuring their APCs, editors-in-chief intentionally committing to improving representation, and centring community voices–meaningful realization of equity within the academic publication space remains a distant goal [1, 6, 7]. Recognizing that shifting systems to centre epistemic justice is a continuous commitment, we argue for pathways that can build on existing foundations and when necessary dismantle existing norms and systems. In this commentary, our goal is to envision pathways forward and to instil a sustainable sense of urgency and a continual commitment. Thus, suggested pathways include two categories: immediate efforts that can provide short to medium-term results and ambitious long-term goals we can aspire towards. Global health cannot fully understand, measure, or address the problems it seeks to solve while the production and circulation of knowledge remain structurally uneven; epistemic justice is therefore not a “nice-to-have”, but a prerequisite for better science and better health outcomes.

## Immediate steps to advance epistemic justice in publishing

Many academic journals, including the Swiss School of Public Health (SSPH+) journals, have committed to lowering or waiving APCs for low income and/or lower middle-income countries (LMICs) [1]. Innovators have made inroads by building journals with business and operational models that prioritize epistemic justice and diversity of representation [8, 9]. For example, Journal of Community Systems for Health (JCSH) uses principles of *Fair Open Alliance* which includes transparent ownership structure and where authors retain copyright of their articles, and require no APCs [8]. Some funders (e.g., Canadian Tri-Agencies, Wellcome Trust) require open access publication and provide funds to enable it. However, we argue that journal policies in relation to APC waivers are not sufficient. Journals that only allow low-income country academics to waive APCs incorrectly assume that researchers in middle-income countries can afford APCs, disregarding the unaffordable costs; these processes also add an administrative burden back to LMIC authors leading to self-exclusion from publishing. Furthermore, there are minimal provisions to waive or provide lower APCs for early career scholars, who then must draw on limited university funds or their advisors’ grants to publish. These barriers limit the flow of ideas from the Global South and of younger scholars, effectively shrinking the solution space.

As a first high-level step, publishing houses could commit to lower profit margins in service of access, particularly those reporting profit margins above 30%. This approach will allow for providing lower or waived APC options and increased publication opportunities. It will be essential to be wary of publishers using hybrid models as a way out, without meaningfully addressing APC related challenges. Publishers also need to prioritize access for use by researchers in LMICs. At minimum, journals and their funding institutes should continue to support maintaining gold open access standards in publishing. Publishers can also strengthen partnerships with LMIC academic institutions to enable both publishing opportunities and access. An intermediary step to establishing gold open access at minimum, could be to remove paywalls for LMICs-based institutions, where the need is greatest or provide a meaningful quota of free-to-read articles–so that researchers and students can readily access material without having to constantly request for full text articles or turn to informal workarounds. Many high-income country (HIC) institutions pay a high price for institutional journal access. However, even HIC institutions have started to refuse paying exorbitant prices of publishers. Swiss universities, for example, could not reach an agreement for open access publishing and reading access with Springer Nature by December 2025, even after a year of negotiations [10]. Springer Nature open access fees remain one of the highest in the academic publishing space. A unified stance by HIC institutions, across countries, to arrest the profiteering by publishing houses should be an option to consider as a power balancing act.

We recognize that many solutions we outlined above are, at best, stop-gap solutions which is dependent upon the benefactory nature of the profit-oriented publishers. We are hopeful that the growing chorus of voices will accelerate more ambitious reforms, such as the ones listed below.

## Expanding horizons of epistemic justice

We imagine a publishing ecosystem organized not around profit extraction, but around knowledge as a public good. In this system, authors, reviewers, editors, funders, universities, governments, and libraries form a co-responsible knowledge common—a sustainable knowledge ecosystem in which value flows back into research, learning, and communities rather than upward into corporate margins. A version of this model exists in diamond open access models where access and publishing remain free [11].

In this envisioned system, academic evaluation shifts from quantity to quality. Researchers are assessed on the rigor, originality, and societal relevance of their contributions rather than on publication counts or journal-based metrics. Deep, careful scholarship—including slow science, replication, context-rich, and social justice-oriented research—is rewarded rather than penalized. This vision builds on commitments such as the San Francisco Declaration on Research Assessment (DORA), which challenge universities, funders, and journals to abandon metric-driven evaluation systems that reinforce inequity [12].

Submission processes explicitly recognize epistemic labour. Authors are invited to reflect on how knowledge in a manuscript was generated, whose interpretations are centred, and which forms of expertise are mobilized or excluded. Reflexivity is not a bureaucratic requirement but a core criterion of scholarly quality.

Peer review is treated as skilled labour rather than invisible service. Reviewers are trained through mentored pathways, particularly for early-career scholars, and are formally compensated or credited in ways that meaningfully count towards academic careers [13, 14].

Article processing charges are eliminated or structurally minimized. Publishing is funded through public, institutional, or consortia models, with any surplus reinvested into research infrastructure, editorial training, and multilingual dissemination. Applying for waivers is fully destigmatized—or rendered unnecessary—because access is not conditional on authors’ ability to pay. This model also encourages Global South governments to invest more into supporting and publishing of research in their own countries.

Crucially, proof of concept versions of these visions already exists. Publicly funded, mission-driven journals and platforms—such as *SciELO, Cadernos de Saúde Pública, JCSH and the Bulletin of the World Health Organization*—demonstrate that high-quality, agenda-setting global health scholarship does not require profit-driven models. In our efforts to reach diamond open access standards, we can learn from and build on funding models these platforms have showcased to continue to fund relevant costs for high quality publishing, while rejecting profiteering and inequitable access in the publishing industry.

The challenge before us is not invention, but scale and recognition. Journals, funders, universities, and researchers must collectively refuse the assumption that legitimacy flows from profit, legacy, and being physically located in the Global North. Academic publishing can—and should—be reorganized to serve equity, dignity, and the public good. Dismantling the current system may be ambitious, but continuing to sustain it is a political choice we can no longer afford. As Maya Angelou reminds us, “Do the best you can until you know better. Then when you know better, do better.” We invite academic publishing industry, governance, academic, research, and funding institutions to do better.
